# Determinants of Vietnam's potential for agricultural export trade to Asia-Pacific economic cooperation (APEC) members

**DOI:** 10.1016/j.heliyon.2023.e13105

**Published:** 2023-01-20

**Authors:** Helian Xu, Do Trong Nghia, Nguyen Hoang Nam

**Affiliations:** a School of Economics and Trade, Hunan University, Changsha, 410006, China; b Thai Nguyen University of Economics and Business Administration, Thai Nguyen, 250000, Viet Nam; c Phenikaa University, Hanoi, 12116, Viet Nam

**Keywords:** Potential trade, Agricultural exports, Technical efficiency, Determinants, Vietnam

## Abstract

This study examines determinants of Vietnamese agricultural exports to the APEC and whether there was an export gap between Vietnam and each APEC trading partner in the period 1998–2018, using the stochastic frontier gravity model. The empirical results affirm the consistency of the gravity model for Vietnamese agricultural exports. The new findings imply that the government should concentrate on designing a policy framework to encourage export enterprises to invest more in the technology factor, especially for large and high-demand markets such as the USA, Japan, and Korea. In practice, export enterprises should focus on several key action points. First, find partners to expand the distribution network of agricultural products within the four largest markets. Second, pay attention to updating information on the technical requirements of China, the largest import market. Third, conduct research to build up competitively strategic products that can be exported to potential new markets, such as Russia, Australia, and Malaysia.

## 1 Introduction

APEC was established in November 1989 in the context of fast-increasing economic integration and cooperation among regions and globally in the late 1980s. APEC operates on three pillars of cooperation: (i) trade and investment liberalization through the gradual reduction and elimination of tariff and non-tariff barriers; (ii) implementation of measures to facilitate business, promote trade and create new job opportunities, reduce production and transaction costs, and increase information exchange and business opportunities; and (iii) economic and technical cooperation aimed at supporting human resource development, improving the capacity of member economies toward equal, balanced and sustainable development. The fact of joining APEC was an important basis and springboard for Vietnam to participate in the integration process, including signing a bilateral trade agreement with the USA in 2000 and accession to the World Trade Organization (WTO) in 2007. Recently, the Vietnamese government has made efforts to negotiate and sign a free trade agreement, the European Union–Vietnam Free Trade Agreement (EVFTA), with the European Union (EU). EVFTA is a comprehensive, high-quality agreement that ensures a balance of benefits for both Vietnam and the EU. The EVFTA agreement officially took effect on August 1, 2020, opening great opportunities and prospects to bring Vietnam–EU relations to new heights. The signing and implementation of the EVFTA agreement makes Vietnam a leading partner of the EU in the Association of Southeast Asian Nations (ASEAN) and one of the Asia-Pacific countries with which the EU has the deepest ties in terms of politics, economy and trade, development cooperation, environment, sustainable energy, and national security. EVFTA will be a huge boost for Vietnam's exports, helping to diversify export markets and products—especially agricultural and aquatic products, as well as other products for which Vietnam has clear competitive advantages. APEC is also a place for Vietnam to reach many important bilateral relations agreements, especially with great powers such as China, the USA, Russia, and Japan.

Vietnam's economic growth in the period 1998–2018 was mainly generated from foreign driving forces, such as foreign direct investment (FDI) and the import-export sector. This was the result of policies to attract FDI and promote exports, especially the commitment to removing import and export barriers of the effective free trade agreements. These “external” driving forces benefited foreign investors with advantages in terms of capital, technology, brand names, and global networks. Foreign investors were also able to take advantage of the “internal” forces wisely and effectively. This has led to disproportionate profits between domestic and foreign investors, to the advantage of foreign investors.

Agricultural product exports play an important role in economic development, especially for developing countries like Vietnam. Agricultural export activities have a strong positive impact on developing countries, increasing income for both domestic agricultural enterprises and traders; improving quality at the cultivation, storage, and transportation stages of agricultural production; increasing foreign exchange; and accelerating economic growth.

The aim of this study is to determine the factors affecting the exports of Vietnamese agricultural products to APEC, a particularly important market for Vietnam's economy. Moreover, it examines in depth the export potential of Vietnam's agricultural products to each member of the APEC. Based on empirical research, the study proposes some specific measures to not only increase the contribution of strong influencing factors but also determine potential markets for Vietnamese agricultural products in the future.

In the field of agricultural exports, Vietnam has had some remarkable achievements. In 1998, the total export turnover of agricultural products reached 2.28 billion USD. A decade later (2008), Vietnam's agricultural export turnover had increased nearly fivefold, to 10.06 billion USD; and by 2018, this figure had more than doubled, to 21.77 billion USD (Table 1).

**Table 1:** The value of Vietnam’ agriculture exports to APEC in the period of 1998–2018 Unit: million USD.

To	1998	2000	2002	2004	2006	2008	2010	2012	2014	2016	2018
APEC	929.2	1320.4	1404.1	1985.5	3593.9	5744.7	7148.3	10,746.0	11,107.3	12,612.7	15,378.6
World	2279.8	2478.7	2600.0	3710.0	5953.8	10,059.0	11,819.7	17,316.3	17,992.5	19,169.5	21,766.8

**Fig. 1:**
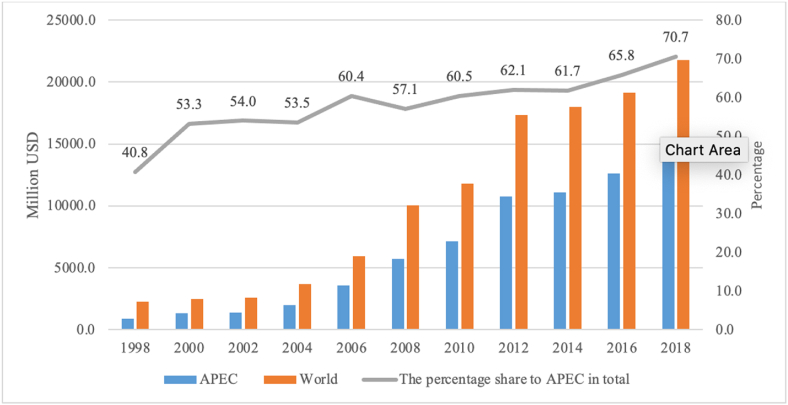
The percentage to APEC in agriculture exports of Vietnam in the period of 1998–2018. *Source*: Author's calculation.

APEC is a very important market for Vietnamese agricultural exports. The export volume to APEC was only around 0.93 billion USD in 1998, accounting for 40.8% of the total value of agricultural exports. By 2008, however, this figure had increased sixfold to 5.74 billion USD, or 57.1% of the total value of agricultural exports; and by 2018, it stood at 15.38 billion USD, or 70.7% of the total (Fig. 1). Thus, the export turnover of Vietnam's agricultural products has increased both in absolute and relative terms, affirming the paramount importance of this market.

Despite the importance of Vietnam's agricultural exports in contributing to the economic growth rate, generating income for households, creating jobs, and helping to reduce the unemployment rate, there are very few studies on this issue. Some scholars have discussed the key products of Vietnam's agriculture (e.g., rice, coffee, tea), such as Vu et al. [1] and Nguyen [2]. Others have researched the export of agricultural products from Vietnam to ASEAN or the EU, including SUN and de Li [3], Pham et al. [4], Tran et al. (2021) [5], and Fan et al. [6]. There has been no research conducted to date on the export of Vietnamese agricultural products to APEC.

This study will contribute to the scholarly literature in many respects. First, it is the first study to discuss the factors affecting the export turnover of Vietnamese agricultural products to APEC. Second, after inheriting the previous research model, this study's model adds two new variables, namely, the institution and the WTO dummy variable. Third, determining the export potential of agricultural products to each country in the bloc should help to clearly define the target market in the medium and long terms. This is a particularly important contribution of this study as compared to all previous research in the same field.

The study endeavors to uncover what determinants affect Vietnam's agricultural export value to APEC countries. It further examines the technical efficiency of Vietnam's agricultural export value to each member economy of APEC and the potential Vietnamese agricultural export value to APEC overall. These are interesting topics not only for policy makers but also for Vietnamese agricultural exporters in practice.

According to the World Bank's WITS database, the major Vietnamese agricultural product groups exported to the APEC are edible fruits and nuts; peel of citrus or melons (code 8); coffee, tea, and spices (code 9); and preparations of vegetables, fruit, nuts, and other parts of plants (code 20). Some of these items have had a strong and steady increase in export turnover over the years, such as cashew nuts, vegetables and fruits, coffee, rubber, and rice, which experienced negative growth in the period 2012–2015 but increased again in the period 2016–2018. Export prices of many agricultural products increased sharply in 2017 and decreased in 2018, revealing the instability in the value of exported Vietnamese agricultural products. Three commodities that experienced an increase in export prices were rice (average export price 501 USD/ton, up 10.7%), tea (average export price 1710.70 USD/ton, up 5%), and cassava/cassava products (average export price 394.90 USD/ton, up 49.8%). In the context of world trade in agricultural products, in which there were many complicated developments—prices fluctuated unpredictably, diplomatic and economic relations between major economies became strained, and protectionism reappeared—Vietnam's agricultural exports maintained positive results in 2018. By maintaining low labor costs and having favorable natural conditions, Vietnam found an advantage in moving toward becoming an export-oriented country. However, one of the major problems affecting Vietnam's agricultural products trade has been the relatively high share of raw materials and low share of processed products [7].

This paper is structured as follows. Section 2 reviews the literature on factors affecting export turnover of agricultural commodities. Section 3 presents data, variables, the model, and the methodology. Section 4 contains an analysis of the empirical results. Section 5 concludes the study and presents some implications.

## 2 Literature review

A close review of the relevant research reveals the knowledge gaps that our research seeks to fill. Few authors have explored the potential trade of concrete goods, such as tea or rice [8,9]. Specifically, Wei et al. [8] indicated that food safety and hygiene standards have an impact on Chinese tea exports; nevertheless, the impact level is unclear because of alternative consuming markets. Kiani et al. [9] and Khan et al. [10] found that geographical distance negatively affects exports and GDP in the case of Pakistan. They also noted that factors positively affecting exports included production, sharing a border, and the GDP of the partner country. Therefore, the authors proposed that a common border with Pakistan would result in higher trade flows.

In the agriculture sector, many researchers used a gravity model to investigate the flow of trade between the home country and key trading partners or economic blocs [11,12]. They concluded that factors such as economy size, market size, openness, population, distance, and exchange rate positively influenced on potential trade.

The most recent study on the export of agricultural products from developing economies to the EU was conducted by Abdullahi et al. [13,14]. This study used a stochastic frontier analysis to investigate the determinants, efficiency, and potential of agricultural products exports from Nigeria to the EU from 1995 to 2019. The empirical results indicated that Nigeria's agri-food exports to the EU were negatively determined by the income of Nigeria and its EU trading countries, bilateral exchange rate, and the EU new member states. Further, Nigeria scores relatively low in terms of the efficiency of its agri-food exports to the EU countries, indicating that Nigeria has a relatively large potential that has not been exploited.

Concerning Vietnam specifically, Thai [15] and Pham et al. [4] investigated bilateral trade between Vietnam and 23 European countries over the 1993–2004 period based on a gravity model. Their studies showed that while factors such as economy size, market size, and exchange rate have a relatively large influence, two variables—geographical and historical distance—have almost no effect on bilateral trade between the two sides. Subsequently, Anh and Thang [16] used a gravity model applied to data derived from the General Department of Customs from 1998 to 2005 to evaluate the determinants affecting trade between Vietnam and ASEAN Plus Three (APT) countries. The empirical results showed that (i) the effect is mainly due to the economic growth (including growth in GDP and GDP per capita) of Vietnam and its partners; (ii) the distance factor seems to affect only exports, not the level of trade concentration of Vietnam with APT; (iii) Vietnam's accession and implementation of commitments with APT are ineffective, with no major impact on Vietnamese trade growth with APT countries identified. This study seems to have been quite successful in applying the gravity model to quantify the determinants affecting the level of commercial concentration.

Nguyen [17] used the gravity model on secondary data from 2001 to 2009 to analyze the impact of the ASEAN and South Korean free trade areas on Vietnamese trade. The research results revealed that with respect to Vietnamese export and import levels, variables in GDP had a positive impact, while distance had a negative effect. Further, the exchange rate variable had a positive impact on exports and a negative impact on imports, while the dummy variables accepted positive coefficients in both export and import models. The results of this study are quite consistent with both theory and practice. Trung [18] studied Vietnam's merchandise exports in the period 2006–2010. The author focused on analyzing specific export turnover and market share for various products long associated with Vietnam, including seafood, rice, and rubber. The author pointed out that lack of capital and of input materials, the complexities of markets, and high transportation costs are determinants affecting Vietnamese merchandise export. The novelty of the study was to focus on some export incentive policies and forecast the export value of some items until 2015.

Additionally, Hung [19] analyzed the consumption of agricultural products in Vietnam before and after joining the WTO on the general basis of the commitments (on tariff barriers and non-tariff barriers). This study also focused on analyzing the consumption policies for Vietnam's agricultural products before and after joining the WTO to propose some recommendations and policies for export of Vietnam's agricultural products in the future. This study has contributed to the consumption of Vietnamese agricultural products in recent years. Pham et al. [4] applied a stochastic frontier analysis to estimate the potential of Vietnamese agriculture exports and further analyze factors concerning the potential of Vietnamese agriculture exports to the EU by a generalized method of moments (GMM) system. The empirical results indicated that such major determinants as the development of financial markets, freedom of trade, technological readiness, and freedom of labor positively affect the potential of Vietnamese agriculture exports.

Recently, Nguyen [2] used the stochastic frontier gravity model to investigate determinants affecting Vietnamese exports of rice and coffee. The empirical results indicated that the effect of “behind-the-border” constraints is statistically significant. Moreover, this study found that there is still potential for Vietnam to increase its exports of rice and coffee with its major trading partners, especially the EU (although not the UK). Therefore, the Vietnamese government should pay more attention to resolving “behind-the-border” constraints to push up export volumes of rice and coffee commodities.

To summarize, although domestic studies apply different approaches and cover a variety of aspects of export activities—export status, export prospects, factors affecting export markets or concentration level trade, the status of the added value of agricultural exports, current export policies, etc.—these studies still largely rely on the status quo and are of a general nature. There is a lack of in-depth studies on factors affecting the export performance of a specific agricultural product and/or group of agricultural products to some important targeted markets.

Compared to these earlier studies, therefore, this study makes several contributions. First, it builds the model to estimate the potential of Vietnamese export trade, which has clear advantages with respect to exporting agricultural products to developed economies, such as the APEC bloc. Second, it adds some independent variables to the model, such as the population, the quality of institution in imported countries, and the dummy variable WTO, for the first time.

## 3 Method

### 3.1 Data and variables

Many studies have used gravity models to evaluate the determinants affecting agricultural products in Vietnam and the world over. Because of differences in time and space, the factors in each given model may or may not be the same. This paper uses the panel data for 19 economies from 2008 to 2018. Table 2 presents all the variables in the empirical model.

**Table 2:** Variables.

Variables	Definition	Source of data	Previous research	Expected mark
GDP	GDP of imported member	World Bank	Wei et al. (2012); Hatab et al. (2010)	+
POP	Population in imported member	World Bank	Wei et al. (2012); Martínez- Zarzoso and Nowak-Lehmann D. (2003)	+/−
DIST	Distance between the two economies	CEPII	Wei et al. (2012); Hatab et al. (2010)	–
^AGRI_LANDi^	The land of agriculture cultivation of i member	World Bank	Pham et al. (2019)	+
^AGRI_VALUEj^	The value of agriculture commodities of j member	World Bank	Authors'	+/−
Exchange_i,t_	Exchange rate of i member at time t	World Bank	Gbetnkom and Khan (2002); Martínez- Zarzoso and Nowak-Lehmann (2003)	–
TECH_i,t_	Technology of i member at time t	The World Economic Forum	Pham et al. (2019)	+
TRADE_FREE	Trade Freedom	The Heritage	Pham et al. (2019)	+
FI-MAR	Financial market development	The World Economic Forum	Pham et al. (2019)	+
LABOR-FREE	Extent of labor freedom	The Heritage	Pham et al. (2019)	+
INS_j,t_	Institution of j member at time t	World Bank	Fayissa and Gill (2015)	+
WTO membership	World Trade Organization membership	World Bank	Anh and Thang (2008)	+

### 3.2 Models and methodology

The classical and new trade theories try to explain the basis for international trade among different nations but do not account for the size of trade flows between countries. However, the gravity model assumes that the volume of trade between countries depends on the distance between countries, GDP, openness, general price level, exchange rate, and the populations of both the exporting and importing countries [20].

The stochastic frontier technique was developed by Aigner, Lovell, and Schmidt [21] and has been widely used to evaluate the performance of companies. Typically, the stochastic frontier model (SFM) assumes a production possibilities frontier that represents the optimal level of production obtained from available fixed inputs. Efficient firms operate on production possibility boundaries, while technically inefficient firms operate within certain limits. It also represents production loss as the difference between actual and potential output, thereby implying that potential output can further expand from a given input.

A SFM is used to calculate the technical efficiency of Vietnamese agriculture exports to the APEC and evaluate the potential exports. To ensure the robustness of the model, this study applies both FEM-cluster and General Least Square (GLS) to analyze the determinants affecting the potential of Vietnamese agricultural product exports to the APEC. Inheriting the model of Pham et al. [4] and developing institution and dummy variables (WTO), the gravity model was applied in equation (1).

(1) $${LnEX}_{ij,t}=\beta_{0}+\beta_{1}{lnGDP}_{j,t}+\beta_{2}{lnPOP}_{j,t}+\beta_{3}{lnDIST}_{i,j}+\beta_{4}{ER}_{i,t}+\beta_{5}{TECH}_{i,t}+\beta_{6}{lnAGRILAND}_{i,t}+\beta_{7}{lnAGRIVALUE}_{j,t}+\beta_{8}{INS}_{j,t}+\beta_{9}{WTO}_{i,j}+\varepsilon_{ij,t}$$

Where: Ln is natural logarithm; i and j indicate Vietnam and trading partner, respectively; t denotes a year t; EX_ij,t_ is the value of Vietnam's agricultural export to member j in year t, measured in millions USD; GDP_j,t_ is the gross domestic product of member j in year t, measured in billions USD; DIST_ij_ is the geographical distance between the capital cities of Vietnam and member j, measured in kilometers; WTO_ij_ is a dummy variable, which equals 1 if Vietnam and the trading partner, economy j, are members of WTO in year t and otherwise equals zero; AGRI_LAND_i,t_ is the agricultural land of Vietnam, measured in hectares; AGRI_VALUE_j,t_ is the value of agricultural sector of member j, measured in millions USD; POPj,t is the total population of member j in year t; ERi,t is the exchange rate of Vietnamdong (VND) and USD; TECH_i,t_ is the technology level of member i at time t; INS_j,t_ is the institution quality of j member at time t.

The study employs the stochastic frontier analysis to estimate Vietnam's export efficiency with the APEC. To this end, the error term is divided into two parts. The first is the pure random term (vij,t); the second is the single-sided error term (*uij,t*), measuring inefficiency and strictly non-negative. Following Battese and Coelli [22], export efficiency is equivalent to the ratio of Vietnamese actual exports to APEC in any given year *t* to the corresponding export when u_ij_ is zero. Therefore, Vietnamese export efficiency to a specific APEC member can be calculated in equation (2):

(2) $${Export\;efficiency}_{ij,t}=\frac{{Actual\;export}_{ij,t}}{{Potential\;export}_{ij,t}}=\frac{\mathrm{Exp}\left(x_{ij,t}\beta +v_{ij,t}-u_{ij,t}\right)}{\mathrm{Exp}\left(x_{ij,t}\beta +v_{ij,t}\right)}=\mathrm{Exp}\;\left({-u}_{ij,t}\right)$$

The export efficiency value varies from zero to unity, with higher export efficiency indicating that export volume is closer to export frontier. A zero value shows the scope to increase actual export to a maximum point, whereas a unity value indicates that actual export equals potential export. The potential export can be calculated by the following equation (3):

(3) $${Potential\;export}_{ij,t}=\frac{{Actual\;export}_{ij,t}}{{Export\;efficiency}_{ij,t}}$$

The study continues to run regression to explore the factors determining the potential of Vietnamese agriculture exports to the APEC on the basis of estimated values of potential agricultural exports on the following equation (4):

(4) $${{LnEX}_{PO}}_{ij,t}=\alpha_{0}+\alpha_{1}{{lnEX}_{PO}}_{ij,t-1}+\alpha_{2}\left({{FI}_{MAR}}_{i,t}\;X\;{{FI}_{MAR}}_{j,t}\right)+\alpha_{3}{{TRADE}_{FREE}}_{j,t}+\alpha_{4}{TECH}_{i,t}+\alpha_{5}{{LABOR}_{FREE}}_{i,t}+\mu_{i,t}$$

In which: EX_PO_ij,t_ is the value of potential Vietnamese agricultural exports to member j in year t, measured in thousands USD; FI_MAR_i,t_ and FI_MAR_j,t_ are respectively the development of financial market of members i and j in year t*.* The values range from the lowest (1) to the highest (7); TRADE_FREE_j,t_ is the freedom of trade in member j in year t. The values range from the lowest (0) to the highest (100). This index is calculated by the following equation (5):

(5) $${{TRADE}_{FREE}}_{j}=\left\{\left(\frac{{Tariff}_{\max}-{Tariff}_{j}}{{Tariff}_{\max}-{Tariff}_{\min}}\right)*100\right\}-{NTB}_{j}$$

where: The max and min levels of tariff show the upper and lower limits of rates. Tariff j indicates the weighted average rate of member j; NTB is non-tariff barriers, with five scales of 20, 15, 10, 5 and 0 respectively; TECH_i,t_ is the technological level of member i in year t. Its values range from the lowest 1 to the highest 7; LABOR_FREE_i,t_ is the labor freedom of member i in year t. The values range from the lowest (0) to the highest (100) based on the following equation (6):

(6) $${Factor\;score}_{i}=50\;\frac{{Factor}_{average}}{{Factor}_{i}}$$

In which the data of member i are calculated in relation to the average of the world and then multiplied by 50. The total score is averaged for country i based on scores of six factors.

Finally, μ_ij,t_ is the error term.

The study employed stochastic frontier analysis to estimate the potential of Vietnamese agriculture exports to the APEC. The Hausman test was then used to compare and determine the suitability of FEM or REM models. Subsequently, the FEM-cluster and GLS approach were used to reaffirm the robustness of the model.

## 4 Results and discussion

There were 399 observations in the dataset. The statistics of all variables used in model 1 and model 4 are presented in Table 3 and Table 4. The results of the Levin-Lin-Chu test demonstrate that all variables are stationary at the original level (Table 5).

**Table 3:** Summary Statistics of model 1.

Variable Name	Obs	Mean	SD	Min	Max
AgriEx	399	3.40e + 08	8.19e + 08	8640.000	7.42e + 09
GDP_j_	399	1.81e + 12	3.54e + 12	9.58e + 09	1.79e + 13
POP_j_	399	1.37e + 08	2.91e + 08	3.19e + 05	1.39e + 09
DIST	399	6839.684	5906.962	873.000	18967.000
Exchange_i_	399	17838.170	3097.412	13268.000	22602.050
Tech_i_	399	3.276	0.547	2.594	5.096
AgriValue_i_	399	232.754	31.434	176.481	281.489
AgriLandA_j_	399	25.850	18.750	1.235	55.183
INSQUA_j_	399	0.472	0.856	−0.943	1.862

**Table 4:** Summary Statistics of model 4.

VarName	Obs	Mean	SD	Min	Max
PO_EX	399	1.01e + 09	1.87e + 09	56959.72	1.49e + 10
FIMAR_ij_	399	19.475	3.768	10.796	33.671
Trade_j_	399	77.376	10.411	34.000	95.000
Lb_i_	399	64.195	4.305	49.700	70.000
Tech_i_	399	3.276	0.547	2.594	5.096

**Table 5:** Levin-Lin-Chu unit root tests.

Variables in model (1)	Adjusted t*	p-value
LAgriEx_ij_	−3.0076	0.0013
LGDP_j_	−2.3414	0.0096
LAgriLand_j_	−3.5818	0.0002
LPOP_j_	−12.4395	0.0000
INSQUA_j_	−3.2377	0.0006
Exchange_i_	52.0657	0.0000
LAgriValue_j_	40.4608	0.0000
**Variables in model (4)**		
LnPO_EX	−1.8902	0.0294
FIMAR_ij_	−6.0776	0.0000
Trade_ij_	−3.7144	0.0001
Labour_i_	−2.9403	0.0016
Tech_i_	21.7873	0.0000

The dataset of this study contains a total observation of 399 (N = 19, T = 21). Table 3 presents the descriptive statistics of the data. The data is balanced and none of the variables deviate substantially from normal distribution. Furthermore, there was no wide range between the minimum and maximum value of our observations except the cases of Distance, AgriValue and AgriLand variables. In model 4, there was a wide range between the minimum and maximum value of FIMAR, trade, and technology variables.

The results of the Levin-Lin-Chu test for stationarity in panel data are shown in Table 5. The test results strongly reject the null hypothesis that all the panels contain a unit root. This means that all variables are stationary at the original level, which is a precondition for avoiding false regression results.

Table 6 presents estimated results run by the stochastic frontier analysis. The empirical results show that the variable of GDP of the importing nation positively influences export turnover of Vietnamese agricultural products. This means that when the scale of the importing economy increases, demand grows for importing agricultural products, especially from developing countries. Similarly, the accession of Vietnam to the WTO and the agriculture value of the importing country is positively associated with the export value of Vietnam's agricultural commodities. This means that when the importing country is a member of the WTO, exports of Vietnamese agricultural products increase. This is completely consistent with the theory and supports the hypothesis. Only the distance between the two countries negatively impacts on the export value of Vietnamese agriculture products. These results are consistent with the practice because the logistics costs of Vietnamese trade are often higher than those of other countries. Moreover, relative to other commodities, agricultural products weigh more and have lower prices. In the long term, increasing the proportion of processed goods in the structure of agricultural product exports is one important means of reducing transportation costs. Because these goods have a high added value, when export turnover increases, the proportion of transportation costs compared to selling prices will decrease.

**Table 6:** Stochastic frontier model.

LAgriEx_j_	Coef.	Std. Err.	z	P > z
LGDP_j_	0.5745	0.1415	4.0600	0.0000
LPOP_j_	0.1642	0.1542	1.0600	0.2870
LDIST_j_	−0.8536	0.1139	−7.4900	0.0000
Exchange_i_	0.0000	0.0000	0.7700	0.4400
Tech_i_	0.4055	0.1608	0.2500	0.8010
LAgriLand_i_	0.7649	0.0708	1.0800	0.2800
LAgriValue_j_	0.1235	0.0742	1.6600	0.0960
INSQUA_j_	−0.0374	0.1128	−0.3300	0.7400
WTO	0.3619	0.1539	2.3500	0.0190
_cons	6.8571	1.9998	3.4300	0.0010

Technical efficiency indicates the ability of an exporter to achieve maximum output from a given set of inputs for a certain level of technology. Measuring technical efficiency is to use input and output, regardless of the price of the above factors. Technical efficiency might be divided into three components such as scale efficiency is a firm's potential to increase productivity to achieve optimum output, an increase in some of the inputs can reduce output, and pure technical efficiency.

Table 7 shows the results of the calculated technical efficiency of Vietnam's agricultural exports to the APEC in the period 1998–2018. Technical efficiencies are low for our data. The average value of technical efficiency is about 27.3% for the whole period, and the range is very large, varying from 5.6% to 84.8%. This finding is consistent with Tran et al. [23], as their research investigated technical efficiency in state rubber farms in Vietnam, and Pham et al. [4], with their study of the technical efficiency of agricultural export to the EU. However, this is not supported by Le [24] and Khai and Yabe [25], both of which examined technical efficiencies in rice production in Vietnam.

**Table 7:** Technical efficiency of agricultural export from Vietnam to the APEC.

TE	1998	1999	2000	2001	2002	2003	2004	2005	2006	2007	2008	2009	2010	2011	2012	2013	2014	2015	2016	2017	2018	1998–2018
APEC	0.115	0.126	0.138	0.151	0.165	0.180	0.195	0.211	0.228	0.245	0.263	0.282	0.301	0.320	0.340	0.360	0.380	0.401	0.421	0.442	0.462	0.273
AUS	0.089	0.103	0.118	0.135	0.153	0.171	0.191	0.212	0.233	0.255	0.277	0.300	0.323	0.347	0.370	0.394	0.417	0.440	0.463	0.486	0.508	0.285
BRN	0.002	0.003	0.004	0.006	0.008	0.010	0.014	0.018	0.023	0.029	0.036	0.044	0.054	0.065	0.077	0.090	0.104	0.120	0.137	0.155	0.174	0.056
CAN	0.057	0.068	0.080	0.094	0.109	0.125	0.142	0.160	0.179	0.199	0.220	0.242	0.264	0.287	0.310	0.333	0.357	0.380	0.404	0.427	0.450	0.233
CHL	0.005	0.006	0.009	0.012	0.016	0.020	0.026	0.032	0.040	0.049	0.059	0.070	0.083	0.097	0.112	0.128	0.145	0.164	0.183	0.204	0.225	0.080
CHN	0.096	0.111	0.127	0.144	0.163	0.182	0.202	0.223	0.245	0.267	0.290	0.313	0.336	0.360	0.383	0.407	0.430	0.453	0.476	0.498	0.520	0.296
HKG	0.115	0.131	0.149	0.167	0.187	0.207	0.228	0.250	0.272	0.295	0.318	0.342	0.365	0.389	0.412	0.435	0.458	0.481	0.503	0.525	0.547	0.323
IDN	0.107	0.122	0.139	0.157	0.176	0.196	0.217	0.238	0.260	0.283	0.306	0.329	0.352	0.376	0.399	0.423	0.446	0.469	0.491	0.514	0.535	0.311
JPN	0.035	0.043	0.052	0.063	0.075	0.088	0.102	0.117	0.134	0.152	0.171	0.190	0.211	0.232	0.254	0.277	0.300	0.323	0.347	0.370	0.394	0.187
KOR	0.058	0.069	0.082	0.095	0.110	0.126	0.144	0.162	0.181	0.202	0.223	0.244	0.267	0.289	0.312	0.336	0.359	0.383	0.406	0.430	0.453	0.235
MEX	0.003	0.004	0.006	0.009	0.011	0.015	0.020	0.025	0.032	0.039	0.048	0.058	0.069	0.081	0.095	0.110	0.126	0.144	0.162	0.181	0.202	0.069
MYS	0.226	0.248	0.270	0.292	0.315	0.338	0.361	0.385	0.408	0.431	0.454	0.476	0.499	0.521	0.542	0.563	0.583	0.603	0.622	0.641	0.659	0.449
NZL	0.034	0.042	0.051	0.061	0.073	0.085	0.099	0.115	0.131	0.149	0.167	0.187	0.208	0.229	0.251	0.273	0.296	0.319	0.343	0.366	0.390	0.184
PER	0.003	0.004	0.006	0.008	0.010	0.014	0.018	0.023	0.029	0.036	0.045	0.054	0.065	0.077	0.090	0.105	0.121	0.138	0.156	0.175	0.195	0.065
PHL	0.202	0.223	0.244	0.266	0.289	0.311	0.334	0.358	0.381	0.404	0.428	0.451	0.473	0.496	0.518	0.539	0.560	0.581	0.600	0.620	0.638	0.425
PNG	0.143	0.161	0.180	0.200	0.221	0.243	0.265	0.287	0.310	0.334	0.357	0.380	0.404	0.427	0.450	0.473	0.495	0.517	0.539	0.560	0.581	0.359
RUS	0.106	0.121	0.138	0.156	0.175	0.195	0.215	0.237	0.259	0.281	0.304	0.328	0.351	0.374	0.398	0.421	0.445	0.467	0.490	0.512	0.534	0.310
SGP	0.750	0.763	0.775	0.787	0.798	0.809	0.819	0.829	0.839	0.847	0.856	0.864	0.872	0.879	0.886	0.892	0.899	0.904	0.910	0.915	0.920	0.848
THA	0.010	0.013	0.017	0.022	0.027	0.034	0.042	0.051	0.062	0.073	0.086	0.100	0.116	0.132	0.150	0.169	0.189	0.209	0.231	0.253	0.275	0.108
USA	0.144	0.162	0.181	0.201	0.222	0.244	0.266	0.289	0.312	0.335	0.358	0.382	0.405	0.428	0.451	0.474	0.497	0.519	0.540	0.561	0.582	0.360

The highest technical efficiency belongs to Singapore (84.8%), followed by Malaysia (44.9%) and the Philippine (42.5%). Notably, Vietnam's key export markets all have low technical efficiency, including the USA (36%), China (29.6%), Korea (23.5%), and Japan (18.7%). However, the technical efficiencies of the USA and China have improved considerably in recent years, up to 58.2% and 52.0% (respectively) in 2018. By contrast, this figure in Japan and Korea is still low: only 39.4% and 45.3% (respectively) in 2018. This forces policymakers to handle pending issues to develop agricultural products for export in the future.

Vietnam's actual agricultural export to the APEC is presented in Table 7. The figures show that the export turnover of Vietnamese agricultural products to APEC has gradually increased over the years, reaching more than 15 billion USD in 2018—of which about 75% of the turnover comes from four major economies. Of these, China is the largest importer, about 47%, equivalent to more than 7 billion USD in 2018, followed by the USA (14.5%), Japan (7%), and Korea (6.5%).

China has become the largest importer for Vietnam's agricultural products for several reasons. First, the purchasing power of 1.4 billion Chinese people for agricultural products has increased, especially for the tropical fruits that are not native to China. Second, the two countries are adjacent and have many international border gates. Third, the two governments allow residents to exchange in the form of small quotas. The USA continues to be the second-largest imported market, reaching nearly 2.3 billion USD in 2018, some valuable and high-quality export products as coffee, rubber, and key fresh fruits like mango, dragon fruit, passion fruit, longan, lychee. This means that exporters effectively exploited the comparative advantages of Vietnamese agricultural products with the biggest economy.

The export turnover of Vietnam's agricultural products to Japan and Korea is nearly the same, around 1 billion USD in 2018. Japan and Korea are both markets with large import and consumption demand for agricultural products. Vietnam is considered a strong exporter in this field, with main products including coffee, vegetables, cashew nuts, and pepper. Some of Vietnam's tropical fruit products, such as dragon fruit, mango, coconut, and lychee, are gradually becoming popular in the market of these two temperate countries.

Despite being a powerhouse in exporting agricultural products in the world, Thailand also has a great demand for importing agricultural products from Vietnam. This presents an opportunity for enterprises to exploit the close geographic proximity of the two countries. Currently, the imported product structure is divided into two distinct groups. First, Thailand imports goods such as coffee and fresh fruit (e.g., litchi and dragon fruit) for domestic consumption. Second, Thailand imports goods for processing, such as cashews, pepper, and chili, and then exports these to the third-country market. In 2018, the total import turnover of Vietnam's agricultural products was 400 million USD.

Potential Vietnamese agricultural exports to the APEC were calculated from the technical efficiency shown in Table 8. These figures indicate that there is still ample room to increase the value of agricultural exports. Specifically, the countries that hold the greatest potential for exported Vietnamese agricultural products are China (13.8 billion USD), the USA (3.85 billion USD), Japan (2.76 billion USD), Korea (2.21 billion USD), Thailand (1.48 billion USD), Philippines (1.36 billion USD), Indonesia (1.13 billion USD), Malaysia (844.8 million USD), Russia (669.9 million USD), and Australia (526.4 million USD), all in 2018. Interestingly, although the import turnover of agricultural products from Vietnam in 2018 is not small, some countries with low potential margins are Singapore (279.3 million USD), Papua New Guinea (42.7 million USD), Peru (23.0 million USD), and Brunei (18 million USD). The gap between the highest and lowest potential is relatively large. These empirical results imply that for these latter four countries, Vietnamese agricultural products do not have much room for export. The cases of Papua New Guinea and Peru are explained by several factors, including low domestic market demand and long geographical distance, which makes it difficult to store fresh fruits and incurs high logistics costs, pushing up selling prices. Despite relative proximity to Vietnam, Singapore and Brunei are fastidious markets with high food safety and hygiene requirements. In 2018, the potential exports to China were 766 times that to Brunei. This gap is explained by the effect of GDP size and the difference in culture factors between Vietnam and trading partners. The potential for Vietnam's agricultural exports to the APEC in Table 9.

**Table 8:** Actual agricultural export of Vietnam to the APEC (mil.USD).

	1998	1999	2000	2001	2002	2003	2004	2005	2006	2007	2008	2009	2010	2011	2012	2013	2014	2015	2016	2017	2018	1998–2018
**APEC**	929.2	1392.0	1320.4	1342.8	1404.1	1705.6	1985.5	2766.9	3593.9	4498.6	5744.7	5241.4	7148.3	9952.6	10746.0	10656.6	11107.3	11335.7	12612.7	14624.4	15378.6	135487.5
AUS	16.2	24.5	34.1	30.0	38.6	55.4	66.9	79.8	85.5	90.0	107.5	102.0	137.1	174.7	177.0	187.7	211.2	225.6	245.5	259.9	267.6	2616.9
BRN	0.02	0.24	0.04	0.01	0.13	0.14	0.46	4.43	2.78	4.43	0.67	3.17	7.88	10.16	9.30	7.44	8.44	7.66	13.57	7.60	3.13	91.7
CAN	52.2	10.5	14.1	13.5	17.2	23.9	41.8	44.8	43.4	46.5	65.6	49.0	70.1	87.2	108.1	135.3	143.7	147.9	169.9	187.9	201.6	1674.2
CHL	0.57	0.01	6.64	0.14	0.05	0.28	2.34	4.10	7.72	3.03	2.46	4.27	11.57	6.04	12.15	16.02	13.62	11.56	11.91	18.30	17.63	150.4
CHN	34.8	197.4	363.9	389.8	321.8	449.9	584.2	899.5	1365.3	1517.4	1749.9	1741.1	2391.8	3951.8	4380.4	4737.6	4198.2	4417.8	5844.1	7418.2	7177.6	54132.4
HKG	155.7	49.7	91.0	58.8	65.5	51.8	49.6	53.9	59.6	89.9	112.5	127.4	193.7	252.0	306.7	297.4	289.2	297.2	231.0	193.3	190.4	3216.1
IDN	303.4	303.4	62.4	91.0	179.1	191.0	34.9	52.5	135.8	482.6	87.3	91.6	468.9	1205.0	682.8	252.7	346.2	396.4	258.9	142.7	602.9	6371.6
JPN	69.2	71.1	105.0	135.0	141.5	121.0	162.0	255.6	286.5	326.1	443.0	327.4	418.2	569.5	657.0	764.5	891.5	1045.0	946.9	1025.9	1085.7	9847.5
KOR	47.6	40.1	41.6	50.7	51.5	74.5	80.3	87.1	129.7	160.6	228.9	180.9	258.2	393.2	489.3	503.2	667.0	640.6	639.6	761.0	999.7	6525.2
MEX	0.3	0.4	1.1	2.8	0.5	0.7	2.0	9.2	13.6	15.1	17.3	16.8	32.9	33.8	92.3	84.2	78.0	42.6	103.4	87.3	75.7	709.7
MYS	18.4	56.1	68.8	64.6	72.8	159.7	146.2	163.4	208.0	260.7	426.7	418.6	465.5	654.8	1055.0	904.2	740.7	639.2	491.8	565.3	556.4	8136.9
NZL	7.8	2.4	2.3	2.1	3.4	3.1	7.3	9.4	6.4	12.6	13.5	14.2	19.2	24.0	25.6	26.9	31.2	32.9	32.5	41.8	41.8	360.3
PER	0.04	0.05	3.17	0.18	0.16	0.31	0.53	1.62	0.96	2.00	9.96	1.91	4.88	6.61	7.88	5.21	5.90	3.93	4.87	3.24	4.47	67.9
PHL	7.0	133.2	106.6	106.6	105.2	121.1	197.0	495.4	475.5	535.3	1253.9	1000.6	1042.2	576.1	626.6	410.7	842.4	677.2	507.8	544.4	870.5	10635.6
PNG	24.78	1.40	2.36	3.00	0.01	1.59	2.98	8.55	6.56	11.21	20.98	19.26	10.25	20.31	20.03	3.72	27.68	76.30	73.16	161.43	24.78	520.4
RUS	19.9	23,6	62.0	110.8	103.2	87.2	125.7	126.9	170.3	181.1	248.7	195.7	262.9	294.7	256.2	323.7	304.4	243.9	270.3	290.8	357.7	4059.6
SGP	93.1	317.3	176.6	120.4	94.9	95.8	81.2	59.1	87.9	108.2	157.9	224.9	336.6	352.4	326.0	410.6	379.2	342.8	252.3	253.3	257.0	4527.5
THA	4.6	53.4	28.0	26.9	22.6	22.5	28.5	32.1	36.5	46.3	87.4	55.7	110.1	174.4	251.1	219.3	289.5	306.4	377.4	396.5	406.6	2975.9
USA	73.6	107.2	150.7	136.4	185.9	245.7	371.7	379.6	472.0	605.7	710.5	666.8	906.1	1165.9	1262.5	1366.3	1639.4	1780.9	2137.9	2265.4	2237.5	18867.6

**Table 9:** The potential for Vietnam's agricultural exports to the APEC (mil.USD).

PO_ EX	1998	1999	2000	2001	2002	2003	2004	2005	2006	2007	2008	2009	2010	2011	2012	2013	2014	2015	2016	2017	2018	1998–2018
APEC	10511.4	13778.8	12337.5	10496.3	9216.4	9413.1	10151.9	13121.5	15256.1	17140.1	19646.5	16065.4	21032.6	27982.3	28936.6	26981.0	26629.9	25758.8	27648.0	30303.9	30336.0	402744.0
AUS	182.7	238.5	288.7	222.8	252.9	323.6	350.4	377.4	367.2	353.4	387.7	339.8	424.1	503.8	477.9	476.5	506.2	512.2	529.6	534.7	526.4	8176.8
BRN	9.8	90.0	10.2	1.6	16.7	13.4	33.8	247.8	121.3	152.6	18.6	71.4	146.1	157.2	121.3	82.7	80.8	63.7	98.9	49.0	18.0	1604.9
CAN	915.3	154.8	175.9	144.1	158.0	191.7	295.0	280.2	242.4	233.3	298.3	202.7	265.7	304.1	349.0	406.3	403.0	388.9	420.8	439.9	447.8	6717.1
CHL	122.7	1.7	752.4	11.9	3.3	13.8	91.2	127.0	193.5	62.1	41.8	61.0	139.9	62.5	108.9	125.2	93.7	70.5	64.9	89.8	78.3	2316.3
CHN	361.8	1775.5	2860.3	2698.4	1977.6	2471.8	2889.7	4031.1	5577.5	5682.6	6039.5	5565.9	7115.4	10989.1	11433.2	11652.0	9765.1	9751.9	12282.1	14888.3	13795.8	143604.7
HKG	1352.9	378.4	611.1	351.7	350.7	250.1	217.2	215.7	218.8	304.6	353.6	372.9	530.8	648.6	744.5	683.4	631.2	618.0	459.0	368.0	348.3	10009.3
IDN	2844.7	2479.4	448.3	578.9	1016.9	974.4	161.1	220.4	521.9	1706.4	285.6	278.5	1330.4	3205.3	1709.4	597.7	776.4	845.4	526.9	277.8	1126.4	21911.9
JPN	1970.4	1647.1	2001.7	2145.6	1896.5	1380.7	1590.1	2178.2	2138.0	2148.6	2596.9	1720.1	1982.3	2451.0	2582.9	2760.8	2972.8	3233.5	2731.4	2770.9	2756.7	47656.3
KOR	817.9	577.6	508.8	531.3	466.5	589.0	558.7	537.7	715.3	797.1	1028.6	740.5	968.7	1359.4	1566.4	1498.7	1856.4	1673.2	1573.9	1770.8	2207.5	22344.0
MEX	91.7	79.6	177.7	326.6	46.2	45.3	100.1	365.9	430.3	385.7	361.4	291.8	477.3	415.2	970.3	765.4	617.5	296.3	638.3	481.2	375.4	7739.2
MYS	81.2	226.4	255.3	221.3	231.2	472.5	404.8	425.0	510.2	604.9	940.2	878.7	933.5	1257.8	1946.6	1606.3	1269.9	1060.0	790.4	882.4	844.8	15843.3
NZL	230.8	56.4	44.7	33.7	46.6	36.3	73.1	81.8	48.5	85.0	80.9	76.1	92.4	105.0	102.2	98.3	105.3	102.9	94.9	114.2	107.1	1816.2
PER	14.2	12.4	569.6	23.0	15.7	22.3	29.4	70.3	32.8	55.0	223.2	35.2	75.3	85.9	87.3	49.7	49,0	28.6	31.3	18.5	23.0	1551.7
PHL	34.7	598.4	436.7	400.9	364.7	389.1	589.0	1384.6	1247.5	1323.6	2932.2	2220.5	2201.9	1162.2	1210.5	761.7	1503.9	1166.3	845.7	878.5	1363.6	23016.2
PNG	173.0	8.6	13.1	15.0	0.1	6.5	11.3	29.7	21.1	33.6	58.8	50.6	25.4	47.6	44.5	7.9	55.9	147.4	135.7	288.2	42.7	1216.8
RUS	188.3	194.5	449.1	709.8	589.6	447.7	583.3	535.8	657.8	643.4	817.3	597.4	749.1	787.0	643.8	768.2	684.7	521.8	551.5	567.9	669.9	12358.0
SGP	124.0	415.9	227.8	153.0	118.9	118.4	99.1	71.2	104.8	127.7	184.4	260.3	386.2	400.9	368.0	460.1	422.0	379.0	277.3	276.7	279.3	5255.1
THA	484.8	4182.6	1675.8	1249.8	828.2	659.0	677.1	626.2	592.3	631.5	1014.0	554.7	951.2	1317.9	1673.1	1298.8	1535.2	1464.9	1637.3	1570.2	1478.0	26102.6
USA	510.4	660.8	830.4	677.0	836.1	1007.6	1397.5	1315.3	1514.8	1808.9	1983.6	1747.2	2237.0	2721.8	2796.9	2881.3	3301.1	3434.0	3958.0	4037.1	3846.9	43503.8

The empirical results of determinants of Vietnam's potential agriculture exports to APEC are presented in Table 10. The authors have run the GLS model to confirm the robustness of the research empirical model. The results show that there is no difference between the FEM-cluster and GLS models, indicating that the regression results provide a sound basis for analysis and forecasting.

**Table 10:** Results of Vietnam's potential agriculture export to APEC.

Variables	Models			GLS
	FEM	REM	FEM-cluster	
LnPO_EX_t-1_	0.373***	1.001***	0.373***	0.0560***
	(0.0463)	(0.00199)	(0.0924)	(0.0102)
FIMAR_ij_	−0.00193	0.00130	−0.00193	−0.000418
	(0.00145)	(0.00148)	(0.00180)	(0.000270)
Trade_j_	0.00312***	−0.000348	0.00312***	0.000418***
	(0.000601)	(0.000533)	(0.000973)	(0.000142)
Tech_i_	0.0189**	−0.0101	0.0189*	0.00376***
	(0.00789)	(0.00780)	(0.00959)	(0.00138)
LB_i_	0.000910	−0.000780	0.000910	0.000157
	(0.000809)	(0.000952)	(0.000921)	(0.000163)
Constant	3.580***	0.0841	3.580***	1.417***
	(0.265)	(0.0653)	(0.558)	(0.0837)
Observations	380	380	380	380
Number of id	19	19	19	19
Hausman test		0.000		
Autocorrelation test		0.0693		
Heteroskedasticity test		0.000		

Standard errors in parentheses.

***p < 0.01, **p < 0.05, *p < 0.1.

Table 10 shows that the variable of potential export at lag 1 is statistically significant at 1% level. Specifically, the volume of potential exports in the previous year affects that in the current year. This can be explained by the fact that Vietnam's agricultural economic sector has grown over time, as has the Vietnamese economy in general. The trade variable, at the statistically significant 1% level, has a positive impact on Vietnam's potential agriculture exports to APEC. This means that the freer the trade, the higher the potential Vietnamese agriculture exports to the APEC. Recently, Vietnam participated in many new generation free trade areas to help reduce both tax and non-tariff barriers. This finding is supported by Riley and Miller [26], Yatsenko et al. [27], Pham et al. [4], Tran et al. [5], and Fan et al. [6] Similarly, the technological readiness of Vietnam positively affects its agriculture exports to the APEC. Almost all enterprises taking part in agricultural commodities have incorporated more modern technologies, increasing the quality of production and processing.

However, in this study, the financial market variable was not shown to be statistically significant. This result is consistent with Arndt and Tarp [28], Managi and Karemera [29], Deichmann et al. [30], Wen et al. [31], and Bui and Nguyen [32]. The labor factor also had no evident impact on the potential of Vietnamese agriculture product exports, likely because there is an abundance of labor in the Vietnamese economy.

## 5 Conclusion

This study used a SFM to calculate the technical efficiency of Vietnam's agriculture exports to the APEC in 1998–2018 period and evaluate its potential exports. Subsequently, it used a FEM-REM model to examine the determinants of Vietnam's potential agricultural exports to APEC members. This study revealed several new findings. First, there is significant potential in Vietnamese agriculture exports to the APEC in general, especially to the major markets of China, the USA, Japan, Korea, and Thailand. Second, there are positive correlations among trade barriers, technological readiness, and potential Vietnamese agriculture exports to the APEC. Third, the technical efficiencies are relatively low and wide-ranging.

To take advantage of opportunities and respond to changing market conditions, the authors propose that the following measures be implemented. First, far-reaching reforms, focused on building policies and institutions, are required to create the best conditions to promote export enterprises to invest more in production, preservation, and post-harvest technologies. Only then will Vietnam's agricultural product exports continue to expand their market share in fastidious countries such as the USA, Japan, and Korea. Even China, Vietnam's largest export market, has recently raised its technical requirements for agricultural products imported from Vietnam. In addition, the Vietnamese government needs to provide updated information to guide the market to help businesses operate more effectively.

Second, there is a need to develop an access plan to the APEC potential markets, such as Russia, Australia, and Malaysia, where the trade potential is quite large and the technical efficiencies are higher than average (i.e., over 50% in the last years of the period studied).

Third, enterprises must adapt their business strategies to the new context, taking competitive pressure as the driving force for innovation and development. Simultaneously, it is necessary to actively seek cooperation with enterprises within the APEC's potential markets (e.g., Russia, Australia, and Malaysia). This is also a good opportunity for Vietnamese businesses to participate more extensively in regional and global supply chains.

Fourth, a focus on high-value official export products to the USA, Japan and Korea would be beneficial. In addition, it is necessary to restructure agricultural fruit products in the direction of being more environmentally friendly and sustainable products Moreover, it would be advantageous to understand the changes in the technical requirements of the Chinese market to minimize the phenomenon of exported agricultural products’ being blocked at the borders, causing significant losses to the export enterprises.

As with other studies, this one has certain limitations. While it analyzes many independent variables, the model does not include the cultural distance factor indicated by Kogut and Singh [33] between Vietnam and its import economies. However, the study also applies a more updated method, GMM, in processing panel data. This leaves room for further studies on potential trade between developing and developed countries.

## Author contribution statement

Helian Xu, Professor: Conceived and designed the experiments; Performed the experiments.

Do Trong Nghia, Ph.D scholar: Analyzed and interpreted the data; Contributed reagents, materials, analysis tools or data; Wrote the paper.

Nguyen Hoang Nam, Ph.D: Contributed reagents, materials, analysis tools or data.

## Funding statement

This research did not receive any specific grant from funding agencies in the public, commercial, or not-for-profit sectors.

## Data availability statement

Data will be made available on request.

## Additional information

Supplementary content related to this article has been published online at [URL].

## Declaration of interest's statement

The authors declare no competing interests.
